# A Solution‐Doped Polymer Semiconductor:Insulator Blend for Thermoelectrics

**DOI:** 10.1002/advs.201600203

**Published:** 2016-09-30

**Authors:** David Kiefer, Liyang Yu, Erik Fransson, Andrés Gómez, Daniel Primetzhofer, Aram Amassian, Mariano Campoy‐Quiles, Christian Müller

**Affiliations:** ^1^ Department of Chemistry and Chemical EngineeringChalmers University of Technology41296GöteborgSweden; ^2^ Physical Sciences & Engineering Division, and KAUST Solar Center (KSC)King Abdullah University of Science and Technology (KAUST)Thuwal23955‐6900Saudi Arabia; ^3^ Department of PhysicsChalmers University of Technology41296GöteborgSweden; ^4^ Institut de Ciència de Materials de Barcelona (ICMAB‐CSIC)Esfera de la UAB08193BellaterraSpain; ^5^ Department of Physics and AstronomyUppsala University75120UppsalaSweden

**Keywords:** conjugated polymers, insulators, molecular dopants, organic thermoelectrics, semiconductors, ternary blend

## Abstract

Poly(ethylene oxide) is demonstrated to be a suitable matrix polymer for the solution‐doped conjugated polymer poly(3‐hexylthiophene). The polarity of the insulator combined with carefully chosen processing conditions permits the fabrication of tens of micrometer‐thick films that feature a fine distribution of the F4TCNQ dopant:semiconductor complex. Changes in electrical conductivity from 0.1 to 0.3 S cm^−1^ and Seebeck coefficient from 100 to 60 μV K^−1^ upon addition of the insulator correlate with an increase in doping efficiency from 20% to 40% for heavily doped ternary blends. An invariant bulk thermal conductivity of about 0.3 W m^−1^ K^−1^ gives rise to a thermoelectric Figure of merit *ZT* ∼ 10^−4^ that remains unaltered for an insulator content of more than 60 wt%. Free‐standing, mechanically robust tapes illustrate the versatility of the developed dopant:semiconductor:insulator ternary blends.

## 1 Introduction

Thermoelectric materials offer great potential for waste heat recovery or as a power source for small scale autonomous electronic components such as radio‐frequency identification tags and sensors,^1^ but also in heat management applications, e.g. when integrated as Peltier elements in a car seat. Current state‐of‐the‐art thermoelectric materials are doped inorganic semiconductors such as bismuth telluride and lead telluride, which are brittle and cumbersome to process and typically contain toxic or scarce elements. Because of their high overall cost, inorganic thermoelectric generators have mostly been employed for extreme applications such as remote power generation of arctic lighthouses and spacecraft.

Instead, organic semiconductors, which can be processed cost‐effectively from solution, represent an interesting alternative. A number of printing and coating techniques can be employed to create the up to millimeter‐thick structures that are needed for thermoelectric applications. The use of polymeric materials is desirable since they permit to adjust the solution viscosity that is required for a particular processing method.^2^ Other advantages of plastics include melt‐processability and superior mechanical properties, the latter of which being a prerequisite for flexible applications. Although a wide range of polymer semiconductors are available today, these materials tend to be of too low molecular weight to provide the desired spectrum of rheological and mechanical properties. Molecular doping, which is needed in the context of thermoelectrics to modulate the charge carrier density, tends to further deteriorate the viscoelastic properties of conjugated polymers and can lead to intractable and brittle materials.^3^, ^4^

The most promising approach to enhance the rheological and mechanical properties of an organic semiconductor is blending with commodity polymers, for which a wide range of molecular weights is readily available. Blends of an organic semiconductor and insulating polymer have been successfully employed for a range of opto‐electronic devices including field‐effect transistors (FETs)^5^, ^6^, ^7^, ^8^, ^9^, ^10^, ^11^, ^12^ and bulk‐heterojunction solar cells,^13^, ^14^ as well as applications such as elastic conductors^15^, ^16^, ^17^ and organic thermoelectrics.^18^, ^19^ In order to maintain a good device performance at low content of the conjugated polymer judiciously chosen processing schemes are necessary. Semi‐crystalline matrix polymers such as polyethylenes and isotactic polystyrene (i‐PS) tend to yield superior results provided that the conjugated polymer crystallizes prior to the insulator.^5^, ^13^ Notably, bulk‐charge transport can be maintained down to a semiconductor content of only 10 wt%, as observed by Kumar et al. using time‐of‐flight photoconductivity measurements.^20^ Alternatively, amorphous polymers such as atactic polystyrene (a‐PS) and poly(methyl methacrylate) can be employed provided that (1) the blend undergoes strong vertical phase separation upon solidification, which is useful for interface devices such as FETs,^6^ (2) phase separation is mitigated through rapid solvent removal in case of fiber spinning,^15^ or (3) the conjugated polymer forms a network of elongated nanowires.^10^, ^18^

So far the use of polymer semiconductor:insulator blends for thermoelectrics has only received limited interest. Recently, Lu et al. examined the thermoelectric properties of poly(3‐butyltiophene):polystyrene (P3BT:PS) blends.^18^ Careful growth of oxygen‐doped P3BT nanofibers that form an interpenetrating network led to an optimal performance for a blend stoichiometry of 60 wt% PS with a Figure of merit $ZT\;=\;\sigma \alpha^{2}T/\kappa$ ∼ 10^−4^, where α is the Seebeck coefficient, *T* is the absolute temperature, and σ and κ are the electrical and thermal conductivity, respectively. One approach to further tune the thermoelectric properties of polymer semiconductor:insulator blends is the addition of a molecular dopant such as 2,3,5,6‐tetrafluoro‐7,7,8,8‐tetracyanoquinodimethane (F4TCNQ), which has been widely studied in conjunction with polythiophenes^11^, ^21^, ^22^, ^23^, ^24^, ^25^, ^26^, ^27^, ^28^, ^29^, ^30^ and is thought to lead to the formation of an ion pair.^31^ In fact, Lu et al. attempted doping of P3BT:PS blends with F4TCNQ but observed that the doping process, which readily occurs in solution, disrupts the formation of P3BT nanowires and results in inhomogeneous films.^18^

In this work, we explore the use of semi‐crystalline poly(ethylene oxide) (PEO) as the matrix polymer in thermoelectric polymer semiconductor:insulator blends. PEO is likely to provide better compatibility with F4TCNQ as well as the conjugated polymer:dopant ion pair due to its higher polarity. We chose to work with the semi‐crystalline conjugated polymer poly(3‐hexylthiophene) (P3HT) because of its good solubility in organic solvents as well as the extensive literature on both doping of P3HT with F4TCNQ^11^, ^21^, ^22^, ^23^, ^24^, ^25^, ^26^, ^27^, ^28^ and P3HT:PEO blends,^32^, ^33^ which aids the selection of appropriate processing protocols to control liquid–liquid phase separation and solid‐state structure formation in F4TCNQ:P3HT:PEO ternary blends. We find that PEO can enable the formation of homogeneous films, leading to a thermoelectric performance that remains unchanged up to an insulator content of more than 60 wt%. Moreover, we observe that ternary blends display significantly enhanced mechanical robustness compared to the notoriously brittle F4TCNQ‐doped P3HT.

## 2 Results and Discussion

**Figure 1:**
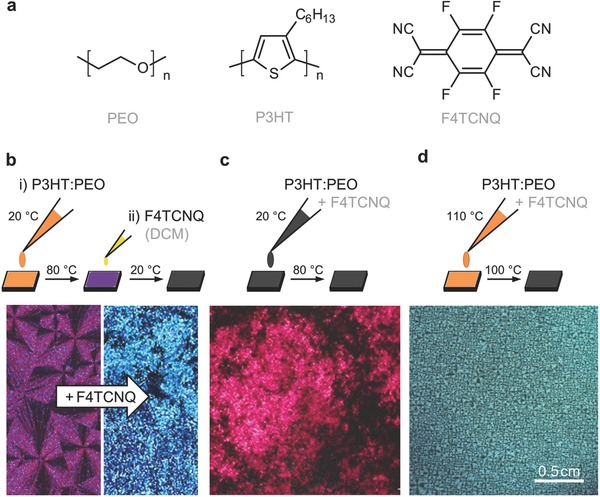
a) Chemical structure of poly(ethylene oxide) (PEO), poly(3‐hexylthiophene) (P3HT) and 2,3,5,6‐tetrafluoro‐7,7,8,8‐tetracyanoquinodimethane (F4TCNQ). b–d) Processing schemes of ternary blends (top) and corresponding optical micrographs (bottom): b) Sequential doping of drop‐cast P3HT:PEO blend films with F4TCNQ:DCM solution; c) Solution‐doping of P3HT:PEO blend solutions with F4TCNQ at room temperature and solvent removal at 80 °C; d) Addition of F4TCNQ to P3HT:PEO blend solutions at 110 °C followed by solvent removal at 100 °C.

In a first set of experiments we explored different processing schemes in order to identify a method that permits the fabrication of homogeneous, F4TCNQ‐doped P3HT:PEO films (**Figure** **1**). Doping of P3HT with F4TCNQ can be carried out either (1) sequentially, which involves film formation followed by treatment with a solution of the dopant in a nonsolvent for the polymer,^34^ or (2) simultaneously, which involves processing of the polymer and dopant from the same solution.^35^ We found that sequential doping –although attractive since it entails a separate film formation and doping step– inevitably deteriorated the quality of P3HT:PEO films due to the good solubility of the matrix polymer in both polar and nonpolar solvents. For instance, treatment with F4TCNQ dissolved in dichloromethane (DCM), which is a nonsolvent for P3HT, resulted in swelling of the PEO matrix that led to pronounced phase‐separation of doped P3HT (Figure 1b).

It is well established that processing of P3HT and F4TCNQ from the same solution is strongly affected by the temperature. One reason is the poor solubility of F4TCNQ in nonpolar organic solvents, which requires temperatures above 80 °C. Moreover, Duong et al. have shown that P3HT and F4TCNQ aggregate at room temperature but dissociate at e.g., 80 °C.^28^ Initially, we explored solution doping of room‐temperature P3HT:PEO blend solutions with F4TCNQ, which appeared deep black due to F4TCNQ:P3HT complex formation. Drop‐casting at 80 °C resulted in strong vertical phase separation of F4TCNQ:P3HT aggregates to the film surface (Figure 1c). In contrast, an increase in the solution temperature to 110 °C led to a visible color change to orange, indicating that the semiconductor and dopant remained well dissolved. We found that drop‐casting at 110 °C permitted the preparation of homogenous films with a thickness of ≈30 μm (Figure 1d). Cross‐polarized optical micrographs revealed a fine microstructure characterized by numerous PEO spherulites. We note that the number of PEO spherulites in doped as compared to pristine P3HT:PEO films increased from ≈60 to 490 spherulites per mm^2^ (Supporting Information, Figure S1). Encouraged by the homogeneous appearance of films drop‐cast at 110 °C we chose to focus all remaining experiments on samples processed according to the scheme detailed in Figure 1d.

**Figure 2:**
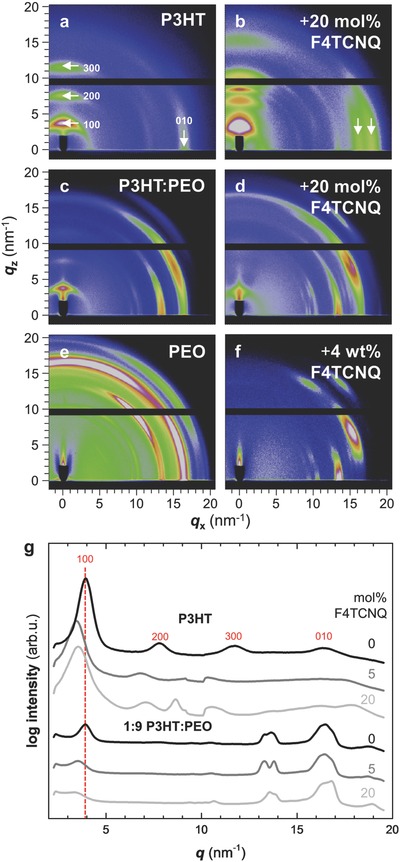
a–f) Grazing‐incidence wide‐angle X‐ray scattering images for a) P3HT, b) P3HT doped with 20 mol% F4TCNQ, c) 1:9 P3HT:PEO, d) 1:9 P3HT:PEO doped with 20 mol% F4TCNQ, e) PEO, and f) PEO with 4 wt% F4TCNQ. g) Diffractograms obtained by integration of GIWAXS images along the azimuthal axis for neat P3HT and P3HT:PEO, as well as doped with 5 and 20 mol% F4TCNQ.

We went on to study the nanostructure of doped semiconductor:insulator films in more detail. Grazing‐incidence wide‐angle X‐ray scattering (GIWAXS) of spin‐coated thin films allowed us to elucidate the impact of F4TCNQ doping on the solid‐state structure of both P3HT and PEO. GIWAXS diffractograms indicate that the out‐of‐plane 100 diffraction of neat P3HT as well as P3HT blended with PEO shifts from 3.9 to 3.5 nm^−1^ upon doping (**Figure** **2**). We explored both weak and strong doping with 5 (not shown) and 20 mol% F4TCNQ, respectively. Moreover, addition of the dopant causes the in‐plane 010 diffraction of P3HT to split into two distinct diffractions close to its original position. These observations are consistent with incorporation of the dopant into the crystal of the semiconductor, leading to the formation of a new organic compound.^22^, ^29^

We employed Rutherford backscattering spectrometry (RBS) to gain insight into the vertical distribution of the semiconductor and matrix polymer in spin‐coated films. The relative intensity of the carbon and sulfur signal (extracted from fits to the RBS spectra) in neat 1:9 P3HT:PEO as well as films doped with 5 and 20 mol% F4TCNQ agreed well with the semiconductor:insulator ratio (Supporting Information, Figure S2). The equivalent width of spectral features associated with sulfur and carbon indicates that the distribution of the two elements is similar throughout the thickness of the film. Since only P3HT contains sulfur we conclude that the here studied samples are characterized by an even depth‐distribution of the semiconductor. This conclusion is corroborated by attempts to fit the recorded spectra with e.g., a bilayer model (Supporting Information, Figure S3), with enhanced surface concentrations of sulfur, which resulted in significantly less accurate fits.

**Figure 3:**
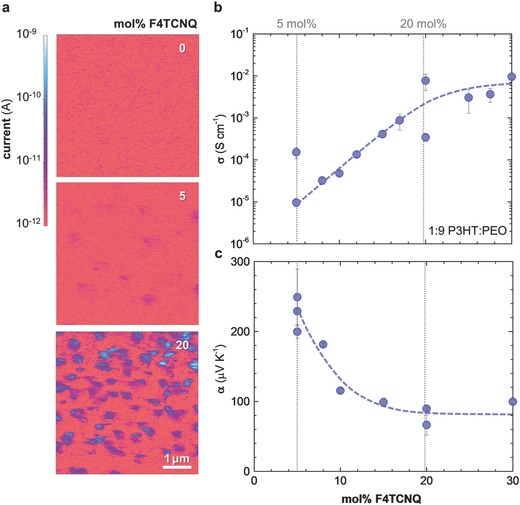
a) Current‐sensing AFM images of thin 1:9 P3HT:PEO blend films: neat blend (top), doped with 5 mol% F4TCNQ (centre), and doped with 20 mol% F4TCNQ (bottom). b) Electrical conductivity σ and c) Seebeck coefficient α of 1:9 P3HT:PEO blend films as a function of F4TCNQ molar fraction relative to the P3HT repeat unit (dashed lines are a guide to the eye).

Current‐sensing atomic force microscopy (CS‐AFM) of spin‐coated films allowed us to study the lateral distribution of the conducting phase. First of all, we observe that blends weakly and strongly doped with 5 and 20 mol% F4TCNQ, respectively, display a higher through‐plane conductivity across the whole scanned surface than undoped 1:9 P3HT:PEO. This observation suggests that – as already indicated by RBS – the conducting phase is continuous throughout the film. Whereas current‐sensing maps of neat 1:9 P3HT:PEO appear featureless, 200 nanometer‐sized domains become visible upon doping that feature a two orders of magnitude higher conductivity than the surrounding material (**Figure** **3**a; Supporting Information, Figure S4). Weakly doped samples contained about 0.5 more highly conducting domains per μm^2^, which increased to 2 domains per μm^2^ for strongly doped blends. We argue that at least part of the F4TCNQ:P3HT co‐crystal phase separates into distinct domains. The remaining material has an approximately one and three orders of magnitude higher conductivity than neat 1:9 P3HT:PEO for weak and strong doping, respectively. Thus, we conclude that in both cases doped films are characterized by highly conducting islands of strongly doped, phase‐separated F4TCNQ:P3HT that are surrounded by moderately conducting material, which mostly consists of PEO that contains more weakly doped P3HT.

In order to investigate the effect of molecular doping on the electrical properties of P3HT:PEO blends we measured both the electrical conductivity σ and the Seebeck coefficient α as a function of F4TCNQ molar fraction (relative to the P3HT repeat unit) for blends with a P3HT:PEO ratio of 1:9 (Figure 3b,c). Duong et al. identified two doping regimes of increasing electrical conductivity below and above 3 mol%, which correspond to two distinct crystal structures that are formed by P3HT and F4TCNQ.^22^ While the crystal structure below 3 mol% strongly resembled that of neat P3HT, a significant change in the diffraction pattern due to the formation of F4TCNQ:P3HT co‐crystals was noted above this critical concentration. Here we focused on the high doping regime because the low semiconductor content of the blend prevented us to measure less conducting samples with an F4TCNQ content lower than 5 mol%. We observed that the electrical conductivity increased from 10^−5^ to 10^−2^ S cm^−1^ when increasing the amount of F4TCNQ from 5 to 20 mol%. Instead, the Seebeck coefficient dropped from 200–250 to 70–90 μV K^−1^, which we rationalize with an increase in charge carrier concentration. Above 20 mol% the electrical properties reached a steady value of σ ∼ 10^−2^ S cm^−1^ and α ∼ 70–100 μV K^−1^. Previously, Duong et al. found that the dopant phase separates above a threshold of 17 mol%, leading to F4TCNQ crystallization due to the saturation of P3HT with F4TCNQ accompanied by a decrease in electrical conductivity.^22^ Interestingly, the here‐studied ternary blends show no decrease in electrical conductivity above 20 mol% F4TCNQ, which we explain with the absence of excess dopant in the conducting phase of the blends. Instead, excess F4TCNQ is likely to be dissolved in the surrounding polar matrix, which is consistent with our observation that the dopant can diffuse into PEO (Supporting Information, Figure S5).

**Figure 4:**
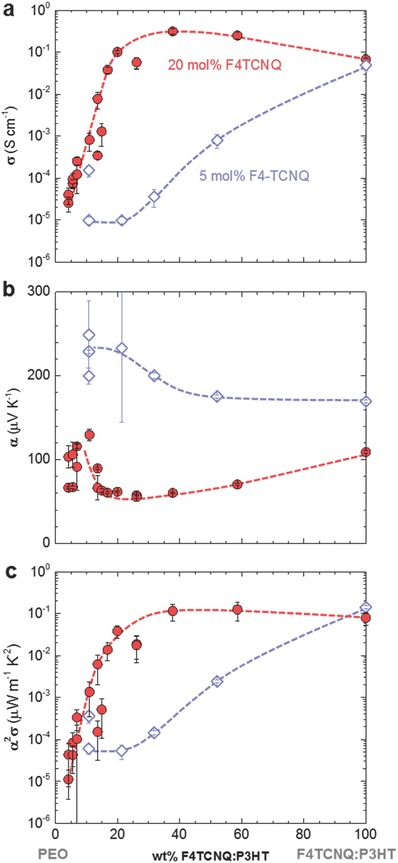
a) Electrical conductivity σ, b) Seebeck coefficient α, and c) power factor α^2^σ of P3HT:PEO blends doped with 5 mol% (blue open diamonds) and 20 mol% F4TCNQ (red filled circles) relative to the P3HT repeat unit (dashed lines are a guide to the eye).

In a further set of experiments we studied the impact of the semiconductor:insulator weight ratio on the thermoelectric properties. We chose to study two series of samples that were weakly and strongly doped with 5 and 20 mol% F4TCNQ, respectively, where the dopant concentration was kept constant relative to the P3HT fraction (**Figure** **4**). In the absence of PEO, weakly and strongly doped P3HT yielded a similar electrical conductivity of about 5 and 7 × 10^−2^ S cm^−1^, respectively. This is in contrast to the work of Duong et al. where the electrical conductivity varied by more than two orders of magnitude for similar doping ratios.^22^ We explain this discrepancy with the difference in processing temperature (100 °C in this work but 80 °C in ref. [22]). Whereas the doping efficiency in films cast at 80 °C remains at 63% independent of the F4TCNQ concentration,^24^ we find that in our samples the doping efficiency decreases from about 40% to 20% when increasing the amount of F4TCNQ from 5 to 20 mol% (cf. Figure 6b). In addition, we argue that weak doping does not disrupt the nanostructure and hence charge‐carrier mobility to the same extent as strong doping.

**Figure 5:**
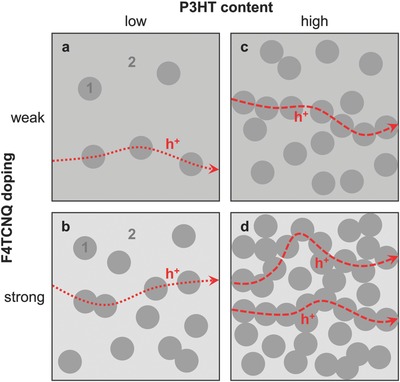
Schematic illustrating the proposed nanostructure of F4TCNQ:P3HT:PEO ternary blends, which consists of F4TCNQ:P3HT rich domains (1) in a PEO rich matrix (2): a low P3HT content results in isolated domains of (1) in case of both a) weak and b) strong doping; a high P3HT content leads to c) only a few poorly connected pathways of (1) in case of weak doping, but d) a percolating network of (1) upon strong doping. The majority of holes (h^+^) select paths that comprise connected domains of (1) and only traverse the matrix (2) in case of insufficient percolation of (1).

For weakly doped blends we observed a rapid decrease in electrical conductivity with increasing PEO content. We propose that conducting pathways are formed by poorly linked F4TCNQ:P3HT domains that are surrounded by the less conducting PEO‐rich matrix (**Figure** **5**b), which reduces the charge carrier mobility. The measured α is the weighted average of contributions from both types of domains, which results in an increase once charge transport through the less doped PEO‐rich matrix starts to dominate (Figure 5a).

Strongly doped samples display a markedly different behavior. Starting from neat F4TCNQ:P3HT, addition of PEO results in a slight increase in σ to 3 × 10^−1^ S cm^−1^ but decrease in α to 60 μV K^−1^ at 38 wt% P3HT:F4TCNQ. We argue that the high amount of F4TCNQ:P3HT domains in strongly doped blends results in a percolating network of the more conducting phase (Figure 5d). As a result, a power factor of 10^−1^ μW m^−1^ K^−2^ can be maintained for an insulator content as high as 62 wt% (Figure 4c). Instead, below 38 wt% F4TCNQ:P3HT the electrical conductivity rapidly decreases by more than four orders of magnitude accompanied by a slight increase in Seebeck coefficient to α > 100 μV K^−1^, leading to a rapid drop in power factor. We explain this behavior with the presence of isolated F4TCNQ:P3HT domains, which are surrounded by the less conducting PEO‐rich matrix (Figure 5c). Whereas σ decreases because of a lower charge carrier mobility, α increases because of the weighted contribution from the less doped PEO‐rich matrix. Comparison with the empirical relation proposed by Glaudell et al. for not‐mobility‐limited systems, i.e., $\alpha \propto \sigma^{-1/4}$,^30^ confirms that blends with low F4TCNQ:P3HT weight fractions of less than 15 wt% suffer from a low charge carrier mobility (Supporting Information, Figure S5).

**Figure 6:**
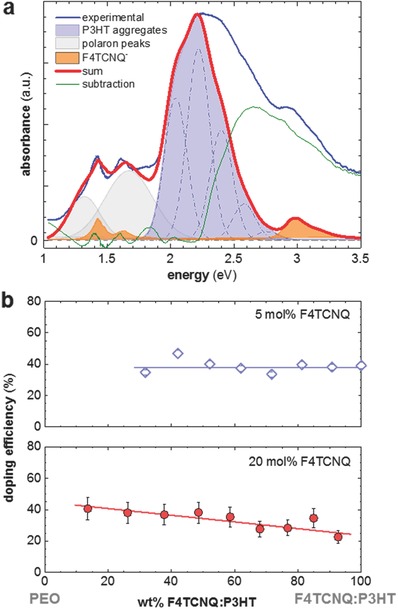
a) Representative optical absorption spectrum of a 1:9 P3HT:PEO blend film doped with 5 mol% F4TCNQ (blue), and decomposition according to Wang et.al into (1) two Gaussians representing the contribution from polaron absorption centred at 1.33 and 1.67 eV, respectively (grey), (2) P3HT aggregate absorption modelled according to Spano et al. (blue),^36^, ^37^ and (3) measured absorption spectrum of the F4TCNQ anion (orange).^24^ The red curve represents the best fit. b) Extracted doping efficiency of P3HT:PEO blends doped with 5 mol% (open diamonds) and 20 mol% F4TCNQ (filled circles) relative to the P3HT:F4TCNQ weight fraction (straight lines are linear fits).

To determine the relationship between doping strength and insulator polymer content for weak and strong doping we extracted the doping efficiency (charge transfer efficiency) of F4TCNQ from UV–vis‐NIR absorption spectra of thin films. We define doping efficiency as the ratio of F4TCNQ anion concentration to total F4TCNQ concentration. **Figure** **6**a shows a representative fit of an absorption spectra fitted as the sum of (1) the F4TCNQ anion signal, (2) two Gaussians corresponding to the polaronic absorption, and (3) a Gaussian model representing the absorption of P3HT aggregates.^24^ The extracted intensity of the F4TCNQ anion was then used to determine the F4TCNQ anion concentration present in the sample through comparison with the total F4TCNQ concentration. To exclude the possibility of F4TCNQ reacting with PEO thus creating additional F4TCNQ anions we recorded UV–vis spectra of F4TCNQ:PEO films that contained 4 wt% of the dopant. We found that only a small amount of dopant reacts with the insulator, resulting in an F4TCNQ anion signal that was several orders of magnitude lower than those measured for the here‐studied ternary blends.

Previously, a doping efficiency of up to 63% was reported for P3HT doped at ambient temperature,^23^, ^24^ which however is known to decrease with processing temperature (cf. decrease in σ and UV–vis anion signal^28^). Hence, for weakly and strongly doped P3HT, which we had deposited at 100 °C, we find a doping efficiency of ≈40% and ≈20%, respectively. Addition of PEO appeared to have no effect on the doping efficiency of weakly doped blends ranging from 9:1 to 3:7 P3HT:PEO (Figure 6b), which is in agreement with the observed invariance in α (cf. Figure 4b; note that σ decreases because of poor connectivity). In contrast, in case of strong doping we observed a linear increase to a doping efficiency of 40% for 1:9 P3HT:PEO, which explains the slight decrease in α for this range of compositions.

We studied the influence of doping on the bulk thermal conductivity of the here investigated ternary blends. For one millimeter thick cold‐pressed samples we observe a thermal conductivity of 0.30 W m^−1^ K^−1^ for undoped 3:7 P3HT:PEO, which is in close agreement with values measured for the neat blend components (**Table** **1**). A corresponding strongly doped sample (3:7 P3HT:PEO; 20 mol% F4TCNQ) yielded a similar thermal conductivity of 0.33 W m^−1^ K^−1^. Based on this value we estimate a Figure of merit of up to *ZT* ∼ 10^−4^ for F4TCNQ:P3HT:PEO ternary blends.

**Table 1:** Bulk thermal conductivity κ and specific heat capacity *C*_p_. The mean and standard deviation for 10 measurements are given

P3HT:PEO	mol% F4TCNQa)	*C*_p_ [J kg^−1^ K^−1^]	**κ** [W m^−1^ K^−1^]
1:0	–	1105 ± 3	0.33 ± 0.01
1:0 b)	20	1246 ± 4	0.26 ± 0.01
3:7	–	1493 ± 7	0.30 ± 0.01
3:7	20	1487 ± 4	0.33 ± 0.01
0:1	–	1446 ± 4	0.36 ± 0.02

^a)^Molar fraction relative to the P3HT repeat unit;

^b)^Doped P3HT was very brittle, which complicated sample preparation and thus may explain the lower thermal conductivity as compared to neat P3HT.

**Figure 7:**
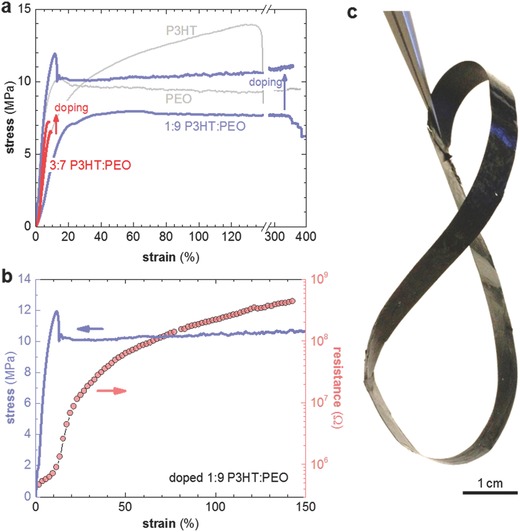
a) Representative tensile stress‐strain curves of drop‐cast films of neat and strongly doped P3HT:PEO blends with a ratio of 1:9 (blue) and 3:7 (red), as well as neat P3HT and PEO (gray); b) electrical resistance (pink) of a 1:9 P3HT:PEO sample (doped with 20 mol% F4TCNQ) during tensile deformation using a manual tensile testing rig; a stress‐strain curve of a similar, independently measured sample is shown in blue; c) image of a tape twisted into a “Möbius Eight”, composed of 3:7 P3HT:PEO doped with 20 mol% F4TCNQ.

Finally, we investigated to which extent the PEO matrix enhances the mechanical properties of the otherwise brittle F4TCNQ‐doped P3HT. We carried out tensile testing of drop‐cast samples that were processed in the same way as samples characterized in Figure 3b,c and Figure 4 (**Figure** **7**a). Neat PEO and P3HT displayed a Young's modulus of 100 and 113 MPa, respectively, which decreased to 80 MPa in case of P3HT:PEO blends (**Table** **2**). Doping resulted in a slightly stiffer material as evidenced by an increase in Young's modulus to about 180 MPa. Note that doped P3HT was too brittle to be subjected to tensile testing. The elongation at break strongly varied with the blend stoichiometry. A high content of the semiconductor resulted in brittle behavior as observed for both neat and strongly doped 3:7 P3HT:PEO. Instead, the ductility of PEO was maintained for both neat and strongly doped samples with a 1:9 P3HT:PEO ratio, which continued to feature an elongation at break of more than 300 %. We recorded the electrical resistance during tensile deformation of a strongly doped 1:9 P3HT:PEO sample (Figure 7b). In the elastic region, i.e., at a strain of less than 10 %, the resistance was not strongly affected. Deformation beyond the yield point resulted in irreversible deformation and an increase in resistance by more than two orders of magnitude at a strain of more than 100 %. We completed our investigation with the preparation of flexible tapes in order to illustrate that the here‐investigated ternary blends permit the fabrication of free‐standing bulk articles (Figure 7c). Evidently, addition of an insulator polymer to a doped polymer semiconductor permits to adjust the mechanical properties, resulting in blends that maintain their thermoelectric performance whilst displaying enhanced robustness.

**Table 2:** Young's modulus *E* and strain at break ε_break_ from tensile deformation. The mean and standard deviation of *E* are based on 3–5 measurements

P3HT:PEO	mol% F4TCNQa)	*E* [MPa]	ε_break_ [%]
1:0	–	100 ± 6	>100
1:0b)	20	–	–
3:7	–	80 ± 22	<10
3:7	20	188 ± 80	<10
1:9	–	83 ± 32	>300
1:9	20	176 ± 18	>300
0:1	–	113 ±58	>300

^a)^Molar fraction relative to the P3HT repeat unit;

^b)^Note that highly doped P3HT was too brittle to be subjected to tensile testing.

## 3 Conclusions

We have shown that up to 62 wt% of the molecular dopant:semiconductor pair F4TCNQ:P3HT can be substituted by the semi‐crystalline polymer insulator PEO without loss of thermoelectric performance. Processing of the F4TCNQ:P3HT:PEO ternary blend from solution allowed the preparation of ≈30 μm thick films with an even nanostructure that consisted of highly doped F4TCNQ:P3HT rich domains in a PEO rich matrix that contained weakly doped P3HT. Ternary blends that contained 62 wt% PEO as well as strongly doped P3HT (20 mol% F4TCNQ) yielded an electrical conductivity of σ ∼ 0.3 S cm^−1^, Seebeck coefficient of α ∼ 60 μV K^−1^ and bulk thermal conductivity κ ∼ 0.33 W m^−1^ K^−1^, which gave rise to a maximum Figure of merit *ZT* ∼ 10^−4^ at room temperature. Corresponding free‐standing tapes displayed improved mechanical properties as compared to the neat dopant:semiconductor pair. We conclude that the use of binder polymers such as PEO opens up a promising avenue that permits to adjust the rheological and mechanical properties of organic thermoelectric materials.

## 4 Experimental Section

*Materials*: Regio‐regular poly(3‐hexylthiophene (P3HT) from Ossila (regioregularity ≈96 %; number‐average molecular weight *M*_n_ ∼ 29 kg mol^−1^; polydispersity index PDI ∼ 2.2), PEO from Polysciences (weight‐average molecular weight *M*_w_ = 5000 kg mol^−1^) and 2,3,5,6‐tetrafluoro‐7,7,8,8‐tetracyanoquinodimethane (F4TCNQ) from TCI Chemicals were used as received without further purification. The solvents ortho‐dichlorobenzene (ODCB; purity > 99 %) and chlorobenzene (CB; purity > 99.5 %) were purchased from Sigma‐Aldrich and DCM (purity > 99 %) was purchased from Fisher Scientific. Size exclusion chromatography (SEC) was carried out on an Agilent PL‐GPC 220 Integrated High Temperature GPC/SEC System in 1,2,4‐trichlorobenzene at 150 °C using polystyrene standards as a reference.

*Sample Preparation*: P3HT and PEO were dissolved at a temperature of 80 °C and a concentration of 20 g L^−1^ in 1:1 mixtures of ODCB:CB. F4TCNQ was dissolved at a concentration of 4 g L^−1^ in DCM for sequential doping and ODCB for solution doping. Then, different ratios of the polymer solutions were mixed to achieve the desired P3HT:PEO ratio, followed by addition of F4TCNQ:ODCB solutions in case of solution doping. The F4TCNQ content was always given as the molar fraction relative to the P3HT repeat unit. Micrometer‐thick films for optical microscopy, electrical conductivity and Seebeck measurements were drop‐cast onto cleaned microscopy glass slides (2.5 × 2.5) cm at the temperatures indicated in Figure 1. In case of sequential doping F4TCNQ:DCM solutions were drop‐cast onto dried films at 80 °C. Thin films were spin‐coated from 110 °C hot F4TCNQ:P3HT:PEO blend solutions onto cleaned and heated quartz substrates for UV–vis, prepatterned glass/ITO for CS‐AFM, or Si/SiO_2_ substrates for GIWAXS (1000 rpm for 90 s followed by 10 s at 3000 rpm). The thickness of drop‐cast and spin‐coated films was determined with a micro‐caliper and a Dektak 150 Profilometer, respectively. A free standing tape of strongly doped 3:7 P3HT:PEO was prepared by drop‐casting from 50 g L^−1^ ODCB:CB solutions onto cleaned microscopy slides, followed by hot‐pressing at 60 °C. Note, that the peak melting temperature of the used PEO grade was 63 °C.

*F4TCNQ Anion Spectrum*: Separate solutions of F4TCNQ and potassium iodide (Sigma‐Aldrich) with a concentration of 1 mol L^−1^ were prepared at 75 °C in acetonitrile (Fisher Scientific; 99.99 % purity). 1.5 molar equivalents of the hot potassium iodide solution were added to the F4TCNQ solution, which turned from yellow to green. The solution was kept at 75 °C for 1 h and then cooled to ambient temperature. One day later a blue precipitate had formed, which was filtered, carefully washed with small amounts of cold acetonitrile and dried under vacuum overnight. The resulting blue powder was dissolved in acetonitrile. Absorption spectra of concentrations ranging from 8 × 10^−6^ to 7 × 10^−5^ mol L^−1^ were taken in order to determine the extinction coefficient ε of the F4TCNQ anion as a function of wavelength.

*Optical Microscopy*: Transmission optical micrographs were recorded with a Zeiss Axio Scope A1.

*UV–Vis Absorption Spectroscopy*: Measurements were performed with a PerkinElmer Lambda 900 spectrophotometer equipped with an integrating sphere. Absorption spectra of doped P3HT:PEO blend films were fitted with two Gaussian peaks (centred at 1.33 and 1.67 eV; full width at half maximum FWHM of 0.29 and 0.42 eV, respectively), a P3HT aggregate Spano model^36^, ^37^ and the F4TCNQ anion spectrum. Peak intensities of the Gaussians and the concentration of the F4TCNQ anion were derived from best fits.^24^

*Current‐Sensing Atomic Force Microscopy (CS‐AFM)*: CS‐AFM images were recorded with an Agilent 5500LS instrument using a Rocky Mountain Nanotechnology solid platinum tip model RMN‐25PT300. The force applied was the same for all the images, 300 nN, which was chosen to ensure a good tip‐sample electrical contact. The current was amplified with a “Resiscope V2” external module attached to the AFM, which applies the bias voltage to the sample while the tip was grounded. The voltage used for all the images where set at the same value, + 1 V DC, current scale was logarithmic.

*Grazing‐Incidence Wide‐Angle X‐Ray Scattering (GIWAXS)*: GIWAXS images were obtained at the D‐line at the Cornell High Energy Synchrotron Source (CHESS) at Cornell University. A synchrotron radiation of a wavelength of 1.155 Å was used for these measurements. A Pilatus 200K detector with pixel size of 172 μm × 172 μm was used to collect X‐rays scattered by the sample at a sample to detector distance of 173.8 mm.

*Rutherford Backscattering Spectrometry (RBS)*: Rutherford backscattering spectrometry was performed using a 2 MeV He^+^ primary beam provided by the tandem accelerator at Uppsala University. Spectra were recorded for particles scattered at 170 degrees with respect to the incident beam direction. The analysis was performed with help of the commercial software package SIMNRA.^38^

*Electrical Characterization*: The electrical resistivity was measured with a four point probe setup from Jandel Engineering (cylindrical probe head, RM3000) using colinear tungsten carbide electrodes with equidistant spacing of 1 mm and held down with a constant weight of 60 g. Seebeck coefficients were measured at 300 K with an SB1000 instrument equipped with a K2000 temperature controller from MMR Technologies using a thermal load of 1–2 K and a constantan wire as an internal reference. Samples of about 1 mm × 4 mm were cut from drop‐cast films and mounted on the sample stage using silver paint (Agar Silver Paint, G302). Measurements were done at a relative humidity of typically not more than 30%; vacuum‐drying had no effect on subsequent Seebeck measurements.

*Bulk Thermal Conductivity Measurements*: Samples for thermal conductivity were prepared by drop‐casting ≈400 mg of material from 50 g L^−1^ ODCB:CB solutions onto cleaned microscopy glass slides, followed by cold pressing of dried material at ambient temperature and 26 kN cm^−2^ to form two pellets with a diameter of 13 mm and a thickness of ≈1.4 mm. The density was determined by measuring the volume and weight of the pellets. A TPS 2500 S Thermal Constants Analyzer from Hot Disk was used to first determine the (volumetric) specific heat capacity and then the thermal conductivity at ambient temperature using sensor 7531 and expanded polystyrene as the surrounding insulating material. An isotropic model was used for data analysis.

*Mechanical Characterization*: Tensile stress‐strain curves of drop‐cast samples were recorded with a DMA Q800 from TA Instruments at room temperature and a constant sample displacement rate of 400 μm min^−1^.

## Supporting information

As a service to our authors and readers, this journal provides supporting information supplied by the authors. Such materials are peer reviewed and may be re‐organized for online delivery, but are not copy‐edited or typeset. Technical support issues arising from supporting information (other than missing files) should be addressed to the authors.

Supplementary
