# Supplementary material for: A Solution‐Doped Polymer Semiconductor:Insulator Blend for Thermoelectrics
Source: Adv Sci (Weinh). 2016 Sep 30;4(1):1600203. doi: 10.1002/advs.201600203 (PMC5238747; doi:10.1002/advs.201600203)
Supplement: Supplementary file 1 — Supplementary [file ADVS-4-0-s001.pdf]

Copyright WILEY-VCH Verlag GmbH & Co. KGaA, 69469 Weinheim, Germany, 2016.

ADVANCED SCIENCE Open Access

# Supporting Information

for *Adv. Sci.,* DOI: 10.1002/advs.201600203

# A Solution-Doped Polymer Semiconductor:Insulator Blend for Thermoelectrics

*David Kiefer, Liyang Yu, Erik Fransson, Andrés Gómez, Daniel Primetzhofer, Aram Amassian, Mariano Campoy-Quiles, and Christian Müller**

Supporting Information

# A Solution Doped Polymer Semiconductor:Insulator Blend for Thermoelectrics

*David Kiefer, Liyang Yu, Erik Fransson, Andrés Gómez, Daniel Primetzhofer, Aram Amassian, Mariano Campoy-Quiles, and Christian Müller**

D. Kiefer, Dr. L. Yu, Dr. C. Müller

Department of Chemistry and Chemical Engineering, Chalmers University of Technology, 41296 Göteborg, Sweden

Email: christian.muller@chalmers.se

E. Fransson

Department of Physics, Chalmers University of Technology, 41296 Göteborg, Sweden

Dr. D. Primetzhofer

Department of Physics and Astronomy, Uppsala University, 75120 Uppsala, Sweden

A. Gómez, Dr. M. Campoy-Quiles

Institut de Ciència de Materials de Barcelona (ICMAB-CSIC), Esfera de la UAB, 08193 Bellaterra, Spain

Dr. L. Yu, Dr. A. Amassian

Physical Sciences & Engineering Division, and KAUST Solar Center (KSC), King Abdullah University of Science and Technology (KAUST), Thuwal 23955-6900, Saudi Arabia

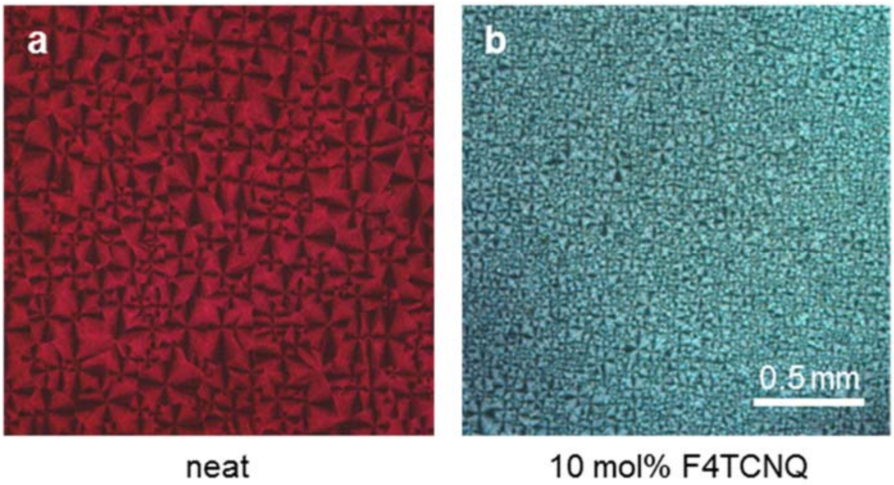


**Figure S1.** Cross-polarized optical micrographs of (**a**) neat 1:9 P3HT:PEO and (**b**) 1:9 P3HT:PEO solution doped with 10 mol% F4TCNQ, cast by following the protocol displayed in Figure 1d. The number of spherulites per surface area increases from 60 spherulites per $mm^2$ for pristine blends to 490 spherulites per $mm^2$ for solution doped blends.

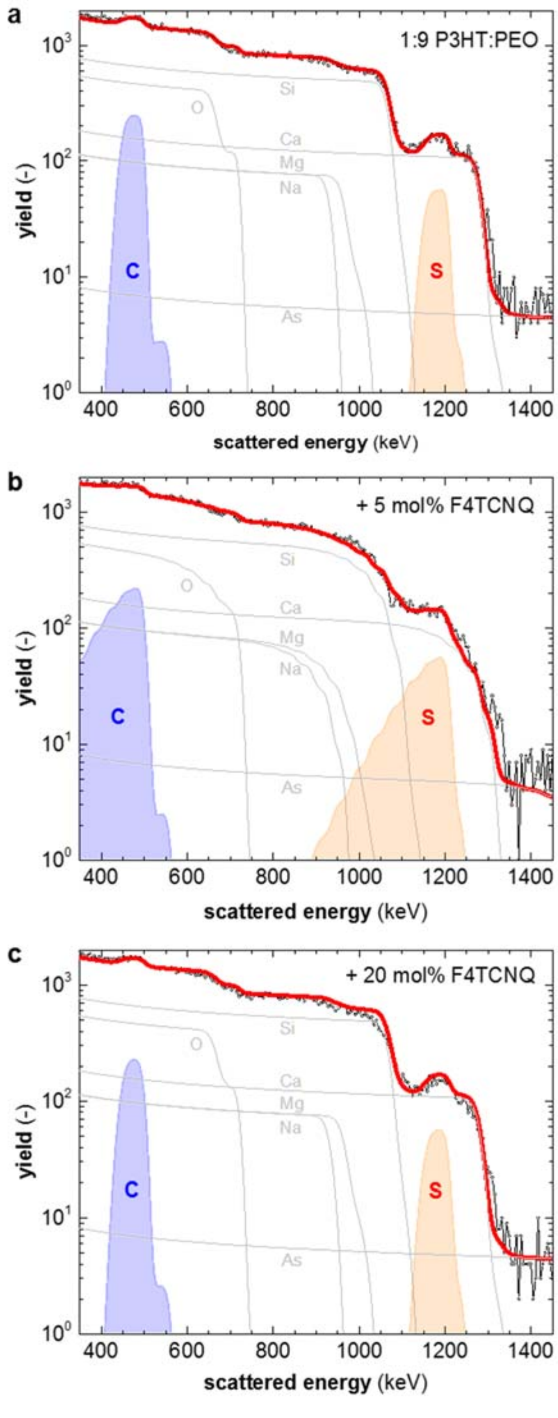


**Figure S2.** Fits to RBS spectra of (**a**) neat 1:9 P3HT:PEO, (**b**) solution doped with 5 mol%, and (**c**) 20 mol% F4TCNQ. The equivalent width of the spectral features of sulfur, associated with the sulfur-containing P3HT, and carbon in all three spectra reveal a similar distribution of the two elements throughout the film and hence an even depth-distribution of P3HT.

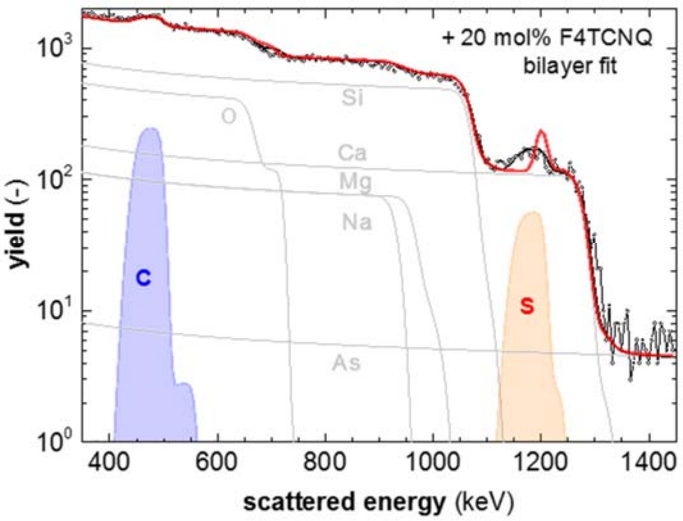


**Figure S3.** Bilayer fit (red) and monolayer fit (black) to an RBS spectrum of 1:9 P3HT:PEO doped with 20 mol% F4TCNQ, showing that fitting the spectra with a bilayer model assuming a surface enhancement of P3HT fails to resemble the measured spectrum.

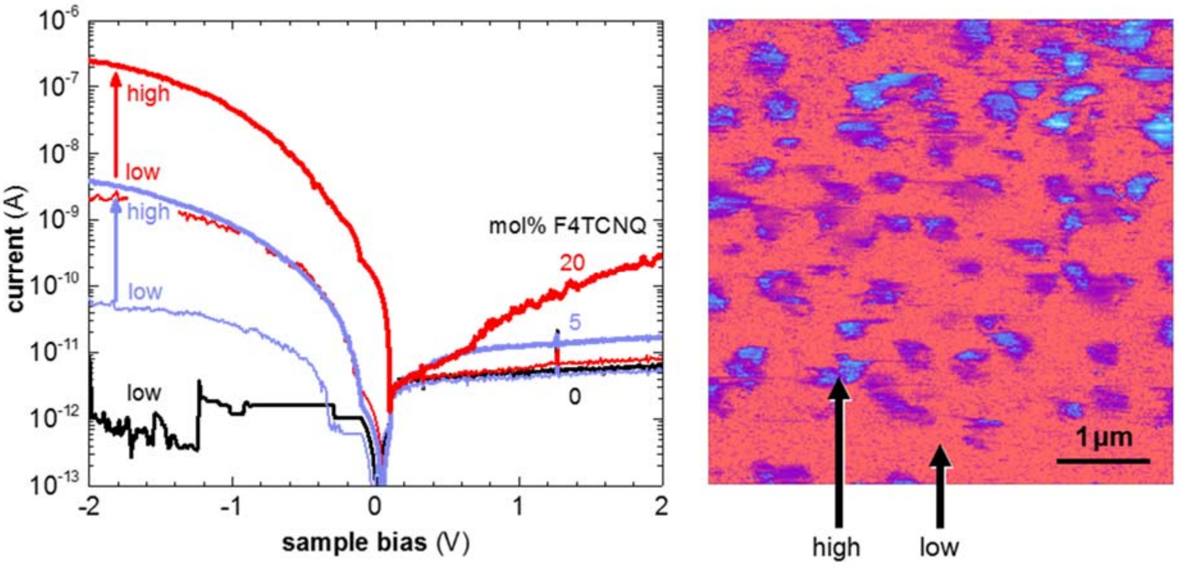


**Figure S4.** Current-Voltage (I-V) curves measured for 1:9 P3HT:PEO doped with 5 mol% (blue) and 20 mol% F4TCNQ (red), as well as for neat 1:9 P3HT:PEO (black). For both types of doped sample the less conducting PEO-rich matrix displays an about two orders of magnitude lower current at e.g. a bias of -2 V as compared to the highly conducting F4TCNQ:P3HT domains, marked with '*low*' and '*high*', respectively (cf. current-sensing AFM image of 1:9 P3HT:PEO doped with 20 mol% F4TCNQ that corresponds to the red I-V curves; arrows indicate areas of low and high conductivity).

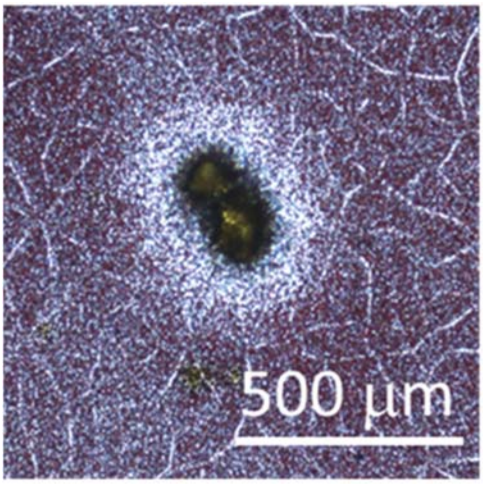


**Figure S5.** Optical micrograph of a F4TCNQ grain placed on top of a drop cast film of 1:9 P3HT:PEO heated to ~65 °C. Note the bright halo surrounding neat F4TCNQ, which we ascribe to diffusion of the dopant into the polar matrix.

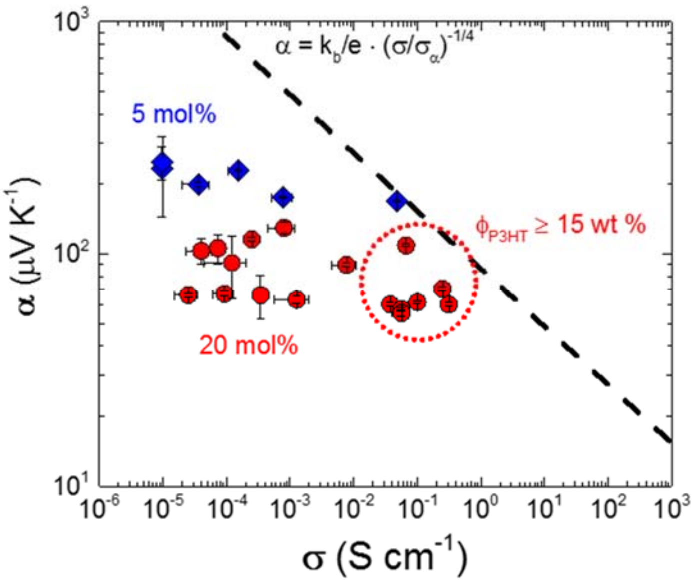


**Figure S6.** Seebeck coefficient (α) as a function of electrical conductivity (σ) for samples doped with 5 and 20 mol% F4TCNQ. The dashed line indicates the empirical trend between α and σ as found by Glaudell et.al. [*Adv. Energy Mater.* **2015**, *5*, 1401072]. We find that only for samples with a P3HT faction of more 15 wt% the measured Seebeck coefficient $\alpha$ and electrical conductivity $\sigma$ approach the empirical trend $\alpha = k_b/e \ \cdot (\sigma/\sigma_0)^{-1/4}$, where $k_b$ is the Boltzmann constant, $e$ is the elementary charge and $\sigma_0$ is a constant set to 1 S $cm^{-1}$. According to Glaudell et al., materials that lie below this trend, i.e. here samples with a P3HT weight fraction of less than 15 wt%, suffer from a too low charge carrier mobility. We ascribe this to a reduced abundance of highly conducting F4TCNQ:P3HT domains and thus insufficient connectivity between them.
